# Transoral Robotic Surgery in the Nordic Countries: Current Status and Perspectives

**DOI:** 10.3389/fonc.2018.00289

**Published:** 2018-07-27

**Authors:** Antti A. Mäkitie, Harri Keski-Säntti, Mari Markkanen-Leppänen, Leif Bäck, Petri Koivunen, Tomas Ekberg, Karl Sandström, Göran Laurell, Mathias von Beckerath, Johan S. Nilsson, Peter Wahlberg, Lennart Greiff, Lena Norberg Spaak, Thomas Kjærgaard, Christian Godballe, Oddveig Rikardsen, Hani Ibrahim Channir, Niclas Rubek, Christian von Buchwald

**Affiliations:** ^1^Department of Otorhinolaryngology – Head and Neck Surgery, University of Helsinki and Helsinki University Hospital, Helsinki, Finland; ^2^Division of Ear, Nose and Throat Diseases, Department of Clinical Sciences, Intervention and Technology, Karolinska Institutet and Karolinska University Hospital, Stockholm, Sweden; ^3^Department of Otorhinolaryngology – Head and Neck Surgery, Oulu University Hospital, Oulu, Finland; ^4^Department of Surgical Sciences, Uppsala University, Uppsala, Sweden; ^5^Department of Otorhinolaryngology – Head and Neck Surgery, Örebro University Hospital, Örebro, Sweden; ^6^Department of Otorhinolaryngology – Head and Neck Surgery, Skåne University Hospital, Lund, Sweden; ^7^Department of Otorhinolaryngology – Head and Neck Surgery, Umeå University Hospital, Umeå, Sweden; ^8^Department of Otorhinolaryngology – Head and Neck Surgery, Aarhus University Hospital, Aarhus, Denmark; ^9^Department of Otorhinolaryngology – Head and Neck Surgery, Odense University Hospital, Odense, Denmark; ^10^Department of Otorhinolaryngology – Head and Neck Surgery, University Hospital North Norway, Tromsoe, Norway; ^11^Department of Otorhinolaryngology, Head and Neck Surgery and Audiology, Rigshospitalet, University of Copenhagen, Copenhagen, Denmark

**Keywords:** head and neck, surgery, management, centralization, cancer

## Abstract

**Background:** The five Nordic countries with a population of 27 M people form a rather homogenous region in terms of health care. The management of head and neck cancer is centralized to the 21 university hospitals in these countries. Our aim was to gain an overview of the volume and role of transoral robotic surgery (TORS) and to evaluate the need to centralize it in this area as the field is rapidly developing.

**Materials and Methods:** A structured questionnaire was sent to all 10 Departments of Otorhinolaryngology—Head and Neck Surgery in the Nordic countries having an active programme for TORS in December 2017.

**Results:** The total cumulative number of performed robotic surgeries at these 10 Nordic centers was 528 and varied between 5 and 240 per center. The median annual number of robotic surgeries was 38 (range, 5–60). The observed number of annually operated cases remained fairly low (<25) at most of the centers.

**Conclusions:** The present results showing a limited volume of performed surgeries call for considerations to further centralize TORS in the Nordic countries.

## Introduction

The five Nordic countries (Denmark, Finland, Iceland, Norway, and Sweden) with a population of 27 M people form a rather homogenous region in terms of health care. The planning and the treatment for head and neck cancer (HNC) for all patients is centralized to the 21 university hospitals only (1). It is regulated by governmental authorities and organized by the public health care system in a fairly unified manner in all of the countries.

Robotic surgery forms an interesting approach in HNC management, but the technical setting has so far warranted a remarkable financial investment, which has obviously resulted in a limited popularity and shared use of existing robots between several surgical specialties at each institution. Since its first use in transoral surgery in 2005 only a limited number of HNC centers worldwide have so far adopted this treatment modality (2, 3). However, along with the increasing incidence of HPV-related oropharyngeal squamous cell cancer (OPSCC) in the Western world the robotic technology continues to develop, and its indications seem to expand to new areas in the management head and neck tumors. Therefore, many Departments of Otorhinolaryngology—Head and Neck Surgery (ORL - HNS) consider including TORS as an option in their surgical armamentarium.

We performed a survey in the Nordic countries aiming to evaluate the current use of transoral robotic surgery (TORS) at the Departments of ORL - HNS. The results of the study might offer tools for initiatives to centralize this treatment modality or to even consider prospective multicenter studies in order to implement evidence-based TORS.

## Materials and methods

TORS is performed at three (Copenhagen, Århus, Odense) out of the four university hospitals, which manage the HNC treatment in Denmark (5.7 M), at two (Helsinki and Oulu) out of the five in Finland (5.5 M), at one (Tromsoe) out of the four in Norway (5.2 M), and at four (Lund, Örebro, Umeå, Uppsala) out of the seven in Sweden (10 M). Iceland has not yet initiated a TORS programme. The number of inhabitants in these referral areas varies from 0.5 to 2.1 M (total population 27 M).

A structured questionnaire was sent in December 2017 to all 10 Nordic Departments ORL – HNS performing TORS. The following data were recorded: the number of inhabitants in the referral area as well as patients referred for TORS from abroad, date of the start of robotic surgery, number of robots and their model, number of surgeons performing TORS, other specialties using the same robot, availability of the robot, surgical indications, and number of performed surgeries specified for each anatomical subsite.

## Results

The Department of ORL-HNS at the Lund University Hospital has used robotic surgery since 2008. The hospital currently has four robots available, whereas all the other centers have started TORS after 2013 and have only one robot dedicated for their use. Solely the Department of ORL-HNS in Copenhagen has their own robot in contrast to all the other centers, which share operative time slots with one to seven other surgical specialties. Half of the reported centers reported insufficient time slots to utilize the robot. All centers currently use the Da Vinci Si model except Aarhus and Odense University Hospitals, which are using the Xi model. The number of trained robotic surgeons for TORS varies from one to three at each Head and Neck center.

The total cumulative number of performed robotic surgeries by the end of 2017 at the 10 centers was 528 and varied between 5 and 240 per center. The median annual number of robotic surgeries was 38 (range, 5–60). The main indication for TORS was the treatment of various neoplasms (*N* = 423). The remaining indications consisted of diagnostic lingual tonsil resections in cases with head and neck carcinoma with unknown primary (HN-CUP), various benign lesions, and of tonsillectomies for training purposes in the initial learning phase of TORS. One center in Sweden and one in Denmark reported having operated on TORS patients also from abroad.

Table 1 shows the distribution of anatomical subsites for tumor resections in this series. The most frequent resection site was base of the tongue (53%). There were no reported TORS operations performed for laryngeal glottic tumors.

**Table 1:** Distribution of the anatomical subsites for tumor resections in the series of 484 TORS cases in the Nordic countries during the 10-year period between 2008 and 2017.

**Subsite**	**Number (%) of surgeries**
Base of tongue^*^	255 (53)
Other oropharyngeal	179 (37)
Nasopharynx	2 (0.4)
Hypopharynx	11(2)
**Larynx**	
Supraglottic	37 (8)
Glottic	0

* *The number of base of tongue resections includes the lingual tonsil resections performed as part of the HN-CUP diagnostic work up*.

## Discussion

In this study we performed a survey on the performance of robotic head and neck surgery at the Departments of ORL-HNS in the five Nordic countries to be able to consider the need and possibilities to centralize this treatment modality in this area with a total of 27 M people. Therefore, it remains obvious that although the technology has been available for more than 10 years, there are currently only 10 centers out of the 21 Departments of ORL-HNS in the Nordic countries that utilize TORS. Furthermore, it is noteworthy, that in the Baltic countries (Estonia, Latvia, Lithuania; total 6 M people) there are currently no surgical robots available. The present study thus covers the whole Northern Europe. However, along with the increasing numbers of OPSCC in the Western world and the improved technical solutions and new surgical indications, robotic surgery may be popularized even more among the institutions managing HNC.

Chen et al. concluded from their National Cancer Database data (*n* = 877) for adults with OPSCC who had undergone TORS, that high-volume centers have the lowest rates of positive margins and unplanned readmissions (4). Similar results were reported by Cracchiolo et al. (5) in a series of 846 OPSCC cases undergoing TORS: positive margin rates were lower when TORS was performed at a high volume vs. low volume hospital (8.2 vs. 16.7% respectively, *p* = 0.001) (5). It has been suggested that consolidating TORS procedures of early stage OPSCC to create high-volume centers of excellence might be a potential strategy to increase incremental effectiveness and reduce incremental costs (6). For other surgical fields there are reports advocating for the centralization of robotic surgery in order to establish high-volume centers with better training possibilities and consequently more efficient use of operating time and thus reduced costs (7–10). The results of the present survey call for considerations to centralize TORS in the Nordic countries. The observed number of annually operated cases (median 38) was fairly low (<25) at most of the centers i.e., at nine out of the ten university hospitals. This result is obviously slightly biased since two of the ten centers had started their TORS activities shortly before the survey and could thus not report their annual number of operated cases. Both of these centers (Odense and Umeå) have now operated 2–4 patients per month during the past weeks, which will eventually affect the median number of annually operated TORS cases in these areas. At the time of the survey in December 2017 only the Copenhagen University Hospital reported one TORS case performed on average each week (total number of 240 cases since 2013) (11). Furthermore, in Iceland, the Department of ORL-HNS does not utilize a robot and in Norway, only one center (Tromsoe) has adopted this technology in 2016 and has now the experience of 37 surgeries.

Since the introduction of TORS more than a decade ago, the indications for robotic surgery in the head and neck area have expanded from including oropharyngeal tumors to neoplasms of the nasopharynx, hypopharynx, larynx and thyroid, and most recently also skull base and neck dissection. The typical anatomical subsite for the primary tumor in the present series was either in the palatine or lingual tonsils, which are easily accessible with TORS. Half of the TORS procedures involved the base of the tongue, which has been shown also by others (11). This is the result of the increasing number of HNC cases with HPV-related HN-CUP and the paradigm of using robotic surgery in the clinical work up of these cases (12, 13).

Successful TORS for laryngeal neoplasms has been mainly restricted to involve supraglottic lesions, which was also observed in the present series. Supraglottic laryngectomy has become one of the standard TORS approaches and several reports have suggested expanding the use of robotic surgery in the management of laryngeal cancer (14). In comparison with the conventional transoral laser surgery, most TORS surgeons use monopolar diathermy although robotic laser instruments are available. Additionally, the access to larynx has been limited by technical factors (mouth retractors, collision of instruments in narrow spaces). The context of applying TORS for laryngeal surgery is now facing a new era with the first reported experiences of a novel semi-rigid operator-controlled robotic system (Medrobotics Flex system) (15). Furthermore, a recent safety and feasibility trial of the da Vinci Single Port (Intuitive Surgical Inc., Sunnyvale, CA, USA) demonstrates that this flexible single-arm device is safe and feasible in performing TORS to access nasopharynx, oropharynx, larynx, and hypopharyngeal areas (16). The development of these new technologies emphasizes the need to consider centralized management of TORS in the Nordic countries.

The current setting for TORS in general clearly involves certain limitations, some of which are related to the da Vinci Surgical System i.e., instrumental and annual service costs. Nevertheless, the costs may be reduced by sharing the robotic instrument in between specialties. In high-volume centers, the set-up (docking) of robot and placement of the mouth retractor have improved quickly over time. Absence of tactile feedback and limited exposure for certain areas of the upper aerodigestive tract still call for further improvement (17). However, the increasing incidence of HPV-related oropharyngeal cancers and the observed improved outcome after surgical treatment in selected cases form a platform for further development of TORS (18). These factors warrant continuous actions in terms of system development and will obviously change the scene for TORS in the future.

We conclude, that a high volume of surgeries at each TORS center in the Nordic countries is needed to maintain a sufficient level of expertise and quality assurance. We also want to emphasize the potential life-threatening complications that are related to TORS and need to be considered in the learning curve phase at each center. Five out of the ten centers in the present survey reported having experienced major postoperative bleeding episodes (data not shown). Therefore, this study has value in describing the current status with TORS in the Nordic countries. Importantly, it also forms the initial steps in creating a forum between TORS surgeons at the respective centers, which is needed to design multicentre studies. Finally, it emphasizes the need for future reflections on where we are headed in terms of TORS in the field of head and neck surgery.

## Author contributions

All authors (AM, HK-S, MM-L, LB, PK, TE, KS, GL, MvB, JN, PW, LG, LN, TK, CG, OR, HIC, NR, CvB) contributed to the data collection, AM prepared the first version of the manuscript and all authors commented on it and approved the final version.

### Conflict of interest statement

The authors declare that the research was conducted in the absence of any commercial or financial relationships that could be construed as a potential conflict of interest.
